# Food insecurity status and its contributing factors in slums’ dwellers of southwest Iran, 2021: a cross-sectional study

**DOI:** 10.1186/s13690-023-01049-8

**Published:** 2023-03-10

**Authors:** Hassan Joulaei, Parisa Keshani, Zohre Foroozanfar, Sima Afrashteh, Zahra Hosseinkhani, Mohammad Ali Mohsenpour, Ghasem Moghimi, Arash Homayouni Meymandi

**Affiliations:** 1 HIV/AIDS Research Center, Institute of Health, Shiraz University of Medical Sciences, Shiraz, Iran; 2 Health Policy Research Center, Institute of Health, Shiraz University of Medical Sciences, Shiraz, Iran; 3 Department of Public Health, School of Public Health, Bushehr University of Medical Sciences, Bushehr, Iran; 4 Metabolic Diseases Research Center, Research Institute for Prevention of Non-Communicable Diseases, Qazvin University of Medical Sciences, Qazvin, Iran; 5 Student Research Committee, Shiraz University of Medical Sciences, Shiraz, Iran; 6 Department of Clinical Nutrition, School of Nutrition and Food Sciences, Shiraz University of Medical Sciences, Shiraz, Iran; 7 Department of Public Health, School of Health, Shiraz University of Medical Sciences, Shiraz, Iran

**Keywords:** Food security, Slum area, COVID-19, Iran

## Abstract

**Background:**

One major factor causing food insecurity is believed to be poverty. Approximately 20 million Iranians live in slums with a vulnerable socioeconomic context. The outbreak of COVID-19, on top of the economic sanctions against Iran, has increased this vulnerability and made its inhabitants prone to food insecurity. The current study investigates food insecurity and its associated socioeconomic factors among slum residents of Shiraz, southwest Iran.

**Methods:**

Random cluster sampling was used to select the participants in this cross-sectional study. The heads of the households completed the validated Household Food Insecurity Access Scale questionnaire to assess food insecurity. Univariate analysis was utilized to calculate the unadjusted associations between the study variables. Moreover, a multiple logistic regression model was employed to determine the adjusted association of each independent variable with the food insecurity risk.

**Results:**

Among the 1227 households, the prevalence of food insecurity was 87.20%, with 53.87% experiencing moderate and 33.33% experiencing severe food insecurity. A significant relationship was observed between socioeconomic status and food insecurity, indicating that people with low socioeconomic status are more prone to food insecurity (*P* < 0.001).

**Conclusions:**

The current study revealed that food insecurity is highly prevalent in slum areas of southwest Iran. The socioeconomic status of households was the most important determinant of food insecurity among them. Noticeably, the coincidence of the COVID-19 pandemic with the economic crisis in Iran has amplified the poverty and food insecurity cycle. Hence, the government should consider equity-based interventions to reduce poverty and its related outcomes on food security. Furthermore, NGOs, charities, and governmental organizations should focus on local community-oriented programs to make basic food baskets available for the most vulnerable households.

## Introduction

Food insecurity, a condition under which households cannot provide adequate, healthy, and nutritious food, can deleteriously affect their health [1]. This phenomenon can lead to nutrient inadequacy [2], an increased risk of hypertension, diabetes [3], coronary heart disease, and heart attack [4], as well as all-cause and cardiovascular-related mortalities [5]. Based on the report jointly released in 2022, from 2019 to 2021, in southeast Asia, southern Asia, northern Africa, and Sub-Saharan Africa, food insecurity has affected 18.8%, 39.4%, 31.1%, and 60.9% of the population, respectively [6]. Additionally, this complication has affected 42.2% of the population in the Middle East and North Africa (MENA) region, as well as 49% of the households in Iran [7]. Since nutrient deficiency has endangered more than one billion lives, food insecurity has attracted particular attention worldwide [8].

Food insecurity is a multidimensional phenomenon. Poverty in low-income populations is a significant factor causing food insecurity [9]. As household-related factors, household type, female-headed households, single-parent families, the householder’s education, ethnicity, the household’s size, the residential area, household income, reliance on financial assistance, household expenditure, and unemployment can all directly affect food insecurity risk [10, 11]. In addition, the above-mentioned household-related factors indirectly lead to a higher food insecurity risk due to lower socioeconomic status and degrees of poverty [12]. In European cities and suburbs, 10% of the households were at risk of food insecurity due to low income and cannot follow a healthy diet [13]. In Iran, evidence showed that household economic status was the most important factor influencing food insecurity in rural households [14].

Another factor causing food insecurity is international political issues. With the reimposition of economic sanctions on Iran since 2018, food markets in Iran have seen a steep rise in prices while incomes have remained stagnant. As a result, maintaining a healthy and nutritious diet has become more challenging for Iranian households, negatively affecting food security among Iranians [15]. Sanctions have caused unemployment, inflation, and loss of the right to health [16].

On the other hand, the outbreak of COVID-19 has exacerbated poverty and, as a result, food insecurity [17]. It has been estimated that approximately 49 million people would face poverty in 2020 due to the pandemic [18] and that 820 million people would face hunger globally by the end of 2020 [19]. Furthermore, in developing countries, a large proportion of the population’s income depends on informal labor, and these populations have low savings. Thus, during the COVID-19 outbreak, lockdown policies reduced the income of a large population in developing countries, thereby increasing food insecurity. As a result, rural–urban migration has increased [20]. Health-related policies during COVID-19 have impaired Iranian rural farmers’ food security by raising unemployment and lowering income [21]. It was shown that the Iranian rural population faced deteriorated food security and changes in consumption of some food groups [22].

According to official statistics, approximately 20 million Iranians live in slums, with 11 million living in informal settlements and the remainder in the forewarn districts. Shiraz, the capital of Fars province and a metropolitan city in the southwest of Iran, is experiencing an increase in the number of people living in the slums, similar to other major cities in Iran [23, 24]. The socioeconomic context of these areas, on the one hand, and the coincidence of the COVID-19 pandemic with economic sanctions against Iran, on the other, can probably exacerbate the vulnerability of its residents to food insecurity. Therefore, the current study aimed to investigate the prevalence of food insecurity and its contributing factors (such as demographic and socioeconomic factors and covid-19 induced economic shortage) among slum residents of Shiraz.

## Methods and materials

### Participants

Data collection was conducted between January and June 2021. Random cluster sampling was used to select the participants in this cross-sectional study in Shiraz, the capital of Fars province in southwest Iran. Each slum area was treated as a cluster, with three out of the eleven suburban areas randomly chosen. All the regions were considered equal in terms of socioeconomic status. The sample size was determined using a 50% prevalence of food insecurity in the Shiraz slum area population, comprising approximately 200,000 people, and a 95% confidence level. Since the households were selected randomly for the sampling, 25% more households were selected in each block so that in case of reluctance to cooperate, the sampling would not face problems. The sample size was calculated to be 1250 households overall and 415–420 households from each of the three slum areas, with ten blocks randomly selected from each. Eventually, 40–45 households in each block were interviewed.

### Measurements

Face-to-face and door-to-door interviews were conducted with household heads after completing informed consent forms. Some general information within each household was collected, such as the head of the household’s age, education level (illiterate, primary, secondary, or high school, diploma, and college), employment status (unemployed, employed, self-employed, pensioner), the number of family members. Their socioeconomic status was calculated considering possession of 9 specific items, including home, personal vehicle, washing machine, LCD TV, dishwasher, refrigerator, handmade rug, laptop, and microwave. Based on the number of items possessed by households, the socioeconomic status was categorized into three groups, low (3 items or less), moderate (4 to 6 items), and high (more than 7 items) [25]. In addition, they were asked whether they had chronic diseases (at least one of the non-communicable diseases, such as diabetes, cardiovascular disease, kidney disease, and cancer), a vulnerable group member in the household (child under 6, adolescent, disabled member, pregnant, handicapped, and elderly), receive financial help from the charity, the portion of income allocated to food purchase, covid-19-induced poverty (including job loss, reduced income, and reduced food purchase), and marital status. The heads of households completed the validated HFIAS (Household Food Insecurity Access Scale) questionnaire to assess food insecurity [26]. The FAO Indicator Guide was used to score a nine-item HFIAS questionnaire [27]. The results were categorized into mild/moderate and severe to make the results more understandable and more appropriate for interventions for policymakers.

### Statistical analysis

Percentages were used to report the distribution of categorized variables. The chi-square test was used to measure the association between the grouped variables in this study. In addition, multiple logistic regression was used to examine the adjusted relationships (odds ratios and 95% CI) between the study variables (household heads, parents’ education, parents’ job, household size, having a chronic disease, having a member of vulnerable groups, consumption of vegetables, fruits, meat, nuts and legumes, socioeconomic status, cost, food cost to income ratio, government financial assistance, covid-19-induced poverty) and food insecurity.

Variables were included in the model (*p*-value under 0.2 in the univariable analysis) if they significantly contributed to the model’s fitness using the stepwise selection method, and the final model was reported. *P*-value < 0.05 was considered significant. STATA14.0 software (Stata, College Station, TX, USA) was used for all the statistical analyses.

## Results

In 1227 households, the prevalence of food insecurity was 87.20%, with 53.87% experiencing mild and moderate hunger and 33.33% experiencing severe hunger. According to Table 1, 1059 (86.3%) of the household heads were male, and approximately 763 (62.5%) households had four family members. Most mothers (49.2%) and fathers (50.1%) had completed primary or secondary school. Most mothers (95.3%) were housewives, while nearly half the fathers (48.3%) worked. Over half of the households had a chronic disease (66.3%). The main reason behind migration among the participants in this study was the lack of job opportunities in their hometown (92.6%). Only 17% of the households were not included as members of the vulnerable groups. In the present study, 8.7%, 9.6%, 3.4%, 5.7%, 0.8%, and 10.7% of the households consumed vegetables, fruits, red meat, legumes, nuts, and dairy products per day, respectively, which were significantly different between secure and insecure households (*P* < 0.001). In the group with food insecurity, the portion of households headed by mothers was significantly higher than father-headed ones (*P* < 0.001). Confirming that the father’s job closely correlates with food insecurity, the proportion of unemployed fathers in this group was higher than that of the secure group (*P* < 0.001). According to our results, the prevalence of chronic diseases was significantly higher in the insecure group (*P* = 0.012). In addition, the number of vulnerable people in food-insecure families was significantly higher. (*P* = 0.032) (Table 1).

**Table 1:** Characteristics of slums’ dwellers household of South-west Iran, 2021

Variables	Total	Secure	Insecure		*p*-value
			**Mild/Moderate**	**Severe**	
**Head of household**	1227	157	661	409	
Father	1059(86.3)	144(91.7)	593(89.7)	322(78.7)	0.001
Mother	156(12.7)	9(5.7)	62(9.4)	85(20.8)	
Other	12(1.0)	4(2.5)	6(0.9)	2(0.5)	
**Father**^**’**^**s education**	1072	148	593	331	
Illiterate	86(8.0)	8(5.4)	45(7.6)	33(10.0)	0.085
Primary or secondary	537(50.1)	71(48.0)	290(48.9)	176(53.2)	
High school & diploma	370(34.5)	56(37.8)	206(34.7)	108(32.6)	
College	79(7.4)	13(8.8)	52(8.8)	14(4.2)	
**Mother**^**’**^**s education**	1194	153	643	398	
Illiterate	134(11.2)	18(11.8)	56(8.7)	60(15.1)	0.003
Primary or secondary	588(49.2)	64(41.8)	317(49.3)	207(52.0)	
High school & diploma	400(33.5)	60(39.2)	225(35.0)	115(28.9)	
College	72(6.0)	11(7.2)	45(7.0)	16(4.0)	
**Father**^**’**^**s job**	1072	150	594	328	
Workless	132(12.3)	6(4.0)	65(10.9)	61(18.6)	0.001
Worker	518(48.3)	62(41.3)	287(48.3)	169(51.5)	
Self-employed	230(21.5)	39(26.0)	133(22.4)	58(17.7)	
Employed & pensioner	192(17.9)	43(28.7)	109(18.4)	40(12.2)	
**Mother**^**’**^**s job**	1171	150	635	386	
Housewife	1116(95.3)	145(96.7)	607(95.6)	364(94.3)	0.448
Employment	55(4.7)	5(3.3)	28(4.4)	22(5.7)	
**Household size**	1220	158	658	404	
< 4	457(37.5)	57(36.1)	248(37.7)	152(37.6)	0.928
≥ 4	763(62.5)	101(63.9)	410(62.3)	252(62.4)	
**Having chronic disease**	1209	158	648	403	
Yes	802(66.3)	118(74.7)	435(67.1)	249(61.8)	0.012
No	407(33.7)	40(25.3)	213(32.9)	154(38.2)	
**Cause of Migration**	517	57	266	194	
Lack of agriculture land & water	23(4.4)	3(5.3)	9(3.4)	11(5.7)	0.515
Lack of jobs	479(92.6)	53(93.0)	251(94.4)	175(90.2)	
Other	15(2.9)	1(1.8)	6(2.3)	8(4.1)	
**Having vulnerable group**	1213	158	650	405	
Child under 6 years	200(16.5)	28(17.7)	102(15.7)	70(17.3)	0.032
Teenager	391(32.2)	58(36.7)	210(32.3)	123(30.4)	
Both child under 6&Teenager	256(21.1)	27(17.1)	145(22.3)	84(20.7)	
Pregnant, handicap or disabled	78(6.4)	5(3.2)	32(4.9)	41(10.1)	
Elderly	82(6.8)	10(6.3)	50(7.7)	22(5.4)	
No	206(17.0)	30(19.0)	111(17.1)	65(16.0)	
**Vegetables consumption**	1207	156	649	402	
Daily	105(8.7)	39(25.0)	52(8.0)	14(3.5)	0.001
Weekly	743(61.6)	103(66.0)	426(65.6)	214(53.2)	
Monthly	332(27.5)	12(7.7)	162(25.0)	158(39.3)	
Rarely	27(2.2)	2(1.3)	9(1.4)	16(4.0)	
**Fruit consumption**	1222	155	660	407	
Daily	117(9.6)	55(35.5)	52(7.9)	10(2.5)	0.001
Weekly	476(39.0)	77(49.7)	285(43.2)	114(28.0)	
Monthly	532(43.5)	22(14.2)	289(43.8)	221(54.3)	
Rarely	97(7.9)	1(0.6)	34(5.2)	62(15.2)	
**Meat consumption**	1211	155	652	404	
Daily	41(3.4)	28(18.1)	11(1.7)	2(0.5)	0.001
Weekly	326(26.9)	69(44.5)	187(28.7)	70(17.3)	
Monthly	652(53.8)	52(33.5)	376(57.7)	224(55.4)	
Rarely	192(15.9)	6(3.9)	78(12.0)	108(26.7)	
**Legumes consumption**	1202	150	652	400	
Daily	69(5.7)	32(21.3)	27(4.1)	10(2.5)	0.001
Weekly	713(59.3)	104(69.3)	409(62.7)	200(50.0)	
Monthly	387(32.2)	12(8.0)	201(30.8)	174(43.5)	
Rarely	33(2.7)	2(1.3)	15(2.3)	16(4.0)	
**Nuts consumption**	1216	155	653	408	
Daily	10(0.8)	5(3.2)	3(0.5)	2(0.5)	0.001
Weekly	63(5.2)	26(16.8)	25(3.8)	12(2.9)	
Monthly	166(13.7)	45(29.0)	85(13.0)	36(8.8)	
Rarely	977(80.3)	79(51.0)	540(82.7)	358(87.7)	
**Dairy consumption**	1213	155	654	404	
Daily	130(10.7)	57(36.8)	50(7.6)	23(5.7)	0.001
Weekly	726(59.9)	91(58.7)	432(66.1)	203(50.2)	
Monthly	311(25.6)	5(3.2)	151(23.1)	155(38.4)	
Rarely	46(3.8)	2(1.3)	21(3.2)	23(5.7)	

Data reported as number (%)

Table 2 represents a significant relationship between socioeconomic status and food insecurity, suggesting that people with low socioeconomic status are more prone to food insecurity (*P* < 0.001). The share of income allocated to food purchases was higher in the food-insecure group (*P* < 0.001). Over 90% of the households did not receive any charitable assistance in this study, which was significantly associated with food insecurity (*P* < 0.001). During the COVID-19 epidemic, the insecure group had a higher rate of job loss and lower food purchases (*P* < 0.05) (Table 2).

**Table 2:** Association between household food security status and socioeconomic variables, in slums’ dwellers of South-west Iran, 2021

Variables	Total	Secure	Insecure		*p*-value
			**Mild/Moderate**	**Severe**	
**Socioeconomic status**	1172	150	634	388	
Low	952(81.2)	95(63.3)	500(78.9)	357(92.0)	0.001^*^
Moderate	220(18.8)	55(36.7)	134(21.1)	31(8.0)	
**Costs of Living**	1222	157	657	408	
< 1 Million	197(16.1)	15(9.6)	69(10.5)	113(27.7)	0.001^*^
1–2 Million	751(61.5)	68(43.3)	448(68.2)	235(57.6)	
≥ 3Million	274(22.4)	74(47.1)	140(21.3)	60(14.7)	
**Share of income allocated to food purchase**	1207	156	648	403	
More than half	536(44.4)	75(48.1)	285(44.0)	176(43.7)	0.001^*^
One third to Half	429(35.5)	30(19.2)	264(40.7)	135(33.5)	
less than One third	242(20.0)	51(32.7)	99(15.3)	92(22.8)	
**Receiving governmental assistance (4$ per months per person)**	1225	158	658	409	
Yes	1172(95.7)	147(93.0)	633(96.2)	392(95.8)	0.210
No	53(4.3)	11(7.0)	25(3.8)	17(4.2)	
**Receive financial help from charity**	1225	158	659	408	
Yes	115(9.4)	8(5.1)	44(6.7)	63(15.4)	0.001^*^
No	1110(90.6)	150(94.9)	615(93.3)	345(84.6)	
**Covid-19 & job loss**	1214	155	656	403	0.031^*^
Yes	795(65.5)	87(56.1)	440(67.1)	268(66.5)	
No	419(34.5)	68(43.9)	216(32.9)	135(33.5)	
**Covid-19 epidemic reduced their income**	1219	156	657	406	
Yes	872(71.5)	101(64.7)	469(71.4)	302(74.4)	0.076
No	347(28.5)	55(35.3)	188(28.6)	104(25.6)	
**Covid-19 epidemic reduced their food purchasing power**	1222	157	659	406	
Yes	1098(89.9)	132(84.1)	596(90.4)	370(91.1)	0.035^*^
No	124(10.1)	25(15.9)	63(9.6)	36(8.9)	

^*^Significant at 0.05 level

### Socioeconomic factors associated with food insecurity

Table 3 illustrates the multiple logistic regression used for evaluating the relationship between food insecurity and socioeconomic factors. According to our findings, allocating “one-third to half” (OR = 5.62, 95%CI: 3.21–9.83, *P* < 0.001) and “more than half” (OR = 1.95, 95%CI: 1.21–3.13, *P* = 0.005) of the income to food purchases were significantly more affected by food insecurity compared to the group with the income to food purchase ratio of less than one third. Father’s self-employment (OR self-employed/ jobless = 0.29, 95%CI: 0.11–0.79, *P* = 0.016), employment, and retirement (OR employed and pensioner / workless = 0.26, 95%CI: 0.09–0.73, *P* = 0.009), having moderate socioeconomic status (OR moderate/low = 0.0.58, 95%CI: 0.37–0.90, *P* = 0.016), and spending more than 30 million Rials per month (Iran’ currency equal to 110 US$) on living costs (OR ≥ 30 million/ < 10 Million = 0.28, 95%CI: 0.12–0.66, *P* = 0.004) were reported as food insecurity-associated determinants (Table 3).

**Table 3:** Factors associated with food insecurity: Univariable and multivariable analysis

Variables	Crude Odds Ratio (95% CI)	Adjusted Odds Ratio (95% CI)
**Head of household**		
Father	1	1 (*P* = 0.201)^**^
Mother	2.57(1.28–5.15)	0.80 (0.20–3.18)
Other	0.31(0.093–1.05)	0.18 (0.02–1.19)
**Father**^**’**^**s Job**		
workless	1	1 (*P* = 0.042)**
Worker	0.35(0.14–0.82)	0.41 (0.16–1.08)
Self-employed	0.23(0.09–0.56)	0.29 (0.11–0.79)
Employed & pensioner	0.16(0.06–0.40)	0.26 (0.11–0.79)
**Having a chronic disease**		
No	1	1
Yes	1.58(1.08–2.31)	1.58 (0.99–2.52)
**Socioeconomic status**		
Low	1	1
Moderate	0.33(0.22–0.48)	0.58 (0.37–0.90)
**Cost**		
< 1Million	1	1 (*P* < 0.001)**
1-2Million	0.82(0.46–1.48)	1.06 (0.46–2.41)
≥ 3Million	0.22(0.12–0.40)	0.28 (0.12–0.66)
**Share of income allocated to food purchase**		
less than One third	1	1 (*P* < 0.001)^**^
One third to Half	3.55(2.19–5.75)	5.62 (3.21–9.83)
More than half	1.64(1.10–2.43)	1.95 (1.21–3.13)
**Receive financial help from charity**		
No	1	1
Yes	2.08(0.99–4.37)	2.89 (0.66–12.64)
**Covid-19 & job loss**		
No	1	1
Yes	1.57(1.11–2.21)	1.40 (0.85–2.31)
**Covid-19 & reduced food purchases**		
No	1	1
Yes	1.84(1.14–2.97)	1.82 (0.97–3.39)

^**^Global *p*-value

*CI* Confidence interval

## Discussion

This cross-sectional study described food insecurity and its associated factors among Shiraz suburban households in southwest Iran. The findings suggest that the food insecurity prevalence was 87.2% (53.87% were moderately and 33.3% were severely hungry). According to the obtained findings, the portion of more than half of one’s income to food purchases increases the chances for food insecurity. Statistically, self-employment, employment by others, retirement, moderate socioeconomic status, and spending more than 30 million Rials (Iran’s currency equal to 110 US$) per month on family expenses were protective factors against food insecurity.

Our findings highlighted that food insecurity is more prevalent among the slum area residents in the southwest of Iran. Based on previous studies, the prevalence of food insecurity in different regions of Iran ranges from 27.8% (urban residents of Shiraz) to 82% (slums of Kerman) [14, 28], which is due to a recent increase in the dust because of wetlands drying up in the southwest and the shortage of water, agricultural mismanagement and low quality of living standards in the southeast of Iran that has led people to migrate to better-off places with job opportunities, such as Shiraz, and staying in suburban areas [29].

A meta-analysis conducted in Iran in 2004 revealed that the prevalence of mild, moderate, and severe food insecurity is 9.3%, 5.6%, and 3.7%, respectively. However, a similar study conducted in 2015 revealed a 49% prevalence [7, 30]. It shows that the trend of food insecurity has increased recently in Iran. Meanwhile, food security has improved significantly in other developing countries, such as India [31]. Evidently, the prevalence of food insecurity was higher in rural and slum than the urban areas [14, 32–34].

Furthermore, studies imply that the prevalence of food insecurity in developing countries is remarkably higher in developed countries. For instance, the prevalence of food insecurity in Lebanon, Nigeria, Nairobi, and Kampala was 50%, 81%, 87%, and 93%, respectively, while it was 16.5% and 15.9% in Portugal and Canada, respectively [35–40]. This indicates that food insecurity is a concerning issue in developing countries. Noticeably, the tools used in various studies for measuring food insecurity were different; thus, comparing different studies should be done cautiously.

Omidvar et al. clustered MENA countries based on GDP and political stability, showing that the frequency of severe food insecurity was significantly different amongst the clusters. Severe food insecurity was 5%, 13.6%, and 26.7% in rich, stable countries, middle-low income with less political stability, and middle-low income politically unstable countries [18].

The socioeconomic status of the households was the most substantial factor associated with food insecurity in the current study; this finding is consistent with those of previous papers in both developed and developing countries [31, 37, 39, 41–43]. The World Bank reported Iran’s poverty rate for 2019 at 17.80%, showing a 3.8% increase from 2018 [44]. Notably, 81.2% of the studied families were categorized as having low socioeconomic status, and 44.4% spent more than half of their monthly income on food. Food insecurity has been discovered to be a significant issue among low-income households. In slum areas, unlike rural ones, most households were immigrants, and due to insufficient natural resources and land for agriculture, their heads worked as manual workers with low wages. [45]. On the other hand, in this study, some active people were jobless, which could worsen the economic status of their households. The overall unemployment rate for 2021 was 11.46% in Iran, based on World Bank reports [46]. In the slum areas of Ahvaz, southern Iran, 16% of households’ heads were unemployed or job-seekers [45]. Furthermore, the present study showed that female-headed households are more likely to have severe food insecurity than male-headed households, consistent with studies conducted in the United States and Kenya [47, 48]. This gender difference revealed that female-headed households would be the top priority for promoting food security programs. As other countries’ experiences show, governmental equity-oriented interventions are required to combat the socioeconomic roots of food insecurity [47–49].

According to our results, the proportion of the members with chronic diseases in the food-insecure group was significantly higher than that of the secure group, consistent with previous studies [35, 50]. In India, households with a physically disabled family member were twice prone to food insecurity [35]. Dean et al. showed that chronic diseases are strenuously linked to food insecurity and higher healthcare expenditures [51]. Evidently, each 1.0 percentage point enhancement in insurance coverage was associated with a food insecurity reduction of 0.4 percentage points [52]. Assisting people in getting health insurance could start a virtuous cycle of improving consequences for health and healthcare as well as food insecurity [52]. A role for such developments in comprehensive anti-poverty strategies may be recommended by food security improvement following the development of health insurance [53].

Similar to studies from Canada and urban areas of Iran, food insecurity was more prevalent in households headed by females than males [33, 43, 50]. Although these households require additional assistance, our findings showed that more than 90% did not receive enough assistance from charities, which was significantly associated with food insecurity in these people. Charities, non-governmental organizations (NGOs), and governmental organizations could have notable roles in empowering female-headed households. Targeted programs for increasing their knowledge and skills, supporting the production and sale of household products (such as sewing, cooking, baking, handicrafts, and jewelry making), and other income-generating skills, such as child and elderly care, can be suggested for planning and implementation regarding cultural, social, and environmental potentials, via these NGOs [54, 55]. Female-headed families in Iran were the subject of a qualitative investigation revealing numerous difficulties that could pose a serious threat [18]. To reduce household food insecurity, governmental organizations should prioritize training them to adapt their new and multidimensional functions, supplying more financial assistance, and supporting them in raising their social standing.

Previous studies in Iran showed that the sanctions and poor domestic policies had significant negative impacts on Iran’s economy. However, among all households, some vulnerable groups suffered more, and sanctions drastically decreased their welfare [18].

Notably, the coincidence of Iran’s economic sanctions and the COVID-19 epidemic has reduced Iranians’ food-purchasing power, exacerbating food insecurity among the middle and low-socioeconomic populations [15]. On the other hand, it has caused an economic recession in Iran, reducing the government’s ability to support low-income families [15, 56]. According to the obtained results, there was no statistically significant relationship between COVID-19 and unemployment or decreased income; meanwhile, it did have a close to significant association (*P* = 0.058) with the decrease in food-purchasing power. Although mismanagement of agricultural sectors in the supply and distribution of food at reasonable prices despite climate change and lack of water resources could not be ignored, Economic sanctions starting over one year before the onset of the COVID-19 epidemic were effective in disturbing the Iranian’s economic status and reducing their purchasing power. In other MENA region countries such as Lebanon, results showed that after the COVID-19 pandemic, food insecurity was estimated at 36% to 39%, with a 50–70% reduction in the population’s income [57]. Moreover, after the COVID-19 pandemic, half the studied population had a low food consumption score, more than half of the households ate less than two meals per day, and about 70% of them missed their meals to spare food [58].

The most important strengths of the present study are as follows: first of all, the results were representative of the general population due to the sampling method. Second, sample size of the study was considered appropriate to guarantee the study’s power. Third, data collection was done with validated tools and a face-to-face method. Finally, an adjusted odds ratio was calculated to determine the association between food insecurity and its contributing factors, resulting in most confounding effects being adjusted.

One of the most significant limitations of this study was its cross-sectional design, limiting the researchers’ ability to investigate the causal pathways between food insecurity and various risk factors. Furthermore, information on the recent trend of food insecurity could not be provided due to the nature of the study. There was a possibility of selection bias by the people not participating in the study due to personal issues and shame. Furthermore, information was possible bias due to their consideration of drawing attention to themselves or shame about their situation.

## Conclusion

The current study revealed that household food insecurity is highly prevalent in slum areas of southwest Iran. The socioeconomic status of households was the most important determinant of food insecurity among them. Hence, mid-term equity-based governmental interventions should be considered to reduce poverty and its related outcomes on food security. In the short-term, governmental and non-governmental organizations should focus on programs that make basic food baskets available for the most vulnerable households, including female-headed and jobless ones. Given the role of charities in reducing the severity of food insecurity, involving them in local community-oriented programs to combat this issue is recommended.

## Data Availability

The datasets used and analysed during the current study are available from “Dr. Hassan Joulaei” on reasonable request.
